# Pontine myelinolysis with an uncommon manifestation of hallucination: a case report

**DOI:** 10.1590/1980-5764-DN-2023-0068

**Published:** 2024-06-24

**Authors:** Andreia Braga Mota Azzoni, Vitor Maia Arca, Eduardo Sousa de Melo

**Affiliations:** 1 Universidade Federal de Pernambuco, Hospital das Clínicas, Departamento de Neurologia, Recife PE, Brazil.

**Keywords:** Mielinólise Central da Ponte, Alucinações, Delírio, Hiponatremia, Myelinolysis, Central Pontine, Hallucinations, Delirium, Hyponatremia

## Abstract

This report aims to present an elderly woman with persistent delirium after hospitalization for lethargy secondary to hyponatremia. The diagnosis of pontine myelinolysis was made and there were no characteristic neurological manifestations such as pupillary changes or spastic tetraparesis. Hallucinations and personality changes were the clues to the diagnosis and should be considered an atypical manifestation of pontine myelinolysis.

## RESUMO

O objetivo deste relato é apresentar uma idosa que apresentou, após internação por letargia secundária à hiponatremia, *delirium* persistente depois da alta hospitalar. O diagnóstico de mielinólise pontina foi feito após a alta hospitalar e não houve manifestações neurológicas características, como alterações pupilares ou tetraparesia espástica. Alucinações e mudança de personalidade foram as pistas para o diagnóstico e devem ser consideradas como uma manifestação atípica de mielinólise pontina.

## INTRODUCTION

Osmotic demyelination syndrome (ODS) is a condition characterized by non-inflammatory etiology. It is a benign syndrome but can be a fatal condition. Serum osmolality is an etiological factor associated with the condition. It is a rare and preventable syndrome. ODS is the main syndrome that includes the concept of Central Pontine Myelinolysis (CPM) and Extra Pontine Myelinolysis. CPM and EPM share the same physiopathology but differ in the anatomic localization of the injury, in addition to evolving with clinical manifestation differences^1,2^.

Central Pontine Myelinolysis is a rare condition. Loss of myelin in the central portion of the pons is the main cause, and demyelination in extrapontine regions can be present^2^. Patients with hyponatremia and with a history of chronic alcoholism were the first reported, especially with a rapid correction of serum natremia^2^. Another characteristic is the variety of the lesions, even though they are often symmetrical.

The classic disease course is described in two phases. Encephalopathy and seizures are the main symptoms in the first, while the second can lead to bulbar symptoms and flaccid quadriparesis, which may progress to spasticity later. The lesion can extend into the tegmentum, and cranial nerve injuries can happen^3^. Mutism, parkinsonism, dystonia, and catatonia can be atypical manifestations^4^.

The most frequent finding is associated with motor deficits. However, ODS uncommonly can occur with behavioral and neuropsychiatric symptoms. There is rare available literature describing behavioral manifestations (personality changes, labile affect, disinhibition, poor judgment, paranoid delusions, emotional lability, delirium, hallucinations, and catatonia), particularly in lesions that saves central line regions^5^.

Considering these issues, we report an atypical case of CPM with delirium and psychotic symptoms as the main manifestation of the syndrome, in the absence of focal neurological motor deficits.

## CASE REPORT

We described a case of a 71-year-old woman, right-handed, married, and retired teacher. The patient had previous diagnoses of arterial hypertension on treatment with a calcium channel blocker, hypothyroidism on hormone replacement, and anxiety treated with escitalopram. The patient and her family gave full permission to publish this case report.

She was admitted to the emergency department with drowsiness and psychomotor slowing. Initial laboratory tests were performed and infectious screening, in addition to cultures, were negative. Severe hyponatremia was observed (sodium 96 mEq/L). She was admitted to the intensive care unit.

During hospitalization, the serum sodium level was corrected from 96 mMol/L to 103 mMol/L in the first 24 hours of hospitalization. After 48 hours, the sodium level rose to 113 mMol/L and continued to increase over the following days to a maximum of 133 mMol/L after 5 days from the start of correction, without exceeding the maximum limit of 12 mMol/L per day. The level of consciousness improved but she manifested psychomotor agitation, as well as delusions and visual hallucinations. Pharmacological treatment for delirium was performed and the patient had partial improvement with quetiapine. During hospitalization, there were no signs of infection or increased inflammatory tests.

**Figure 1:**
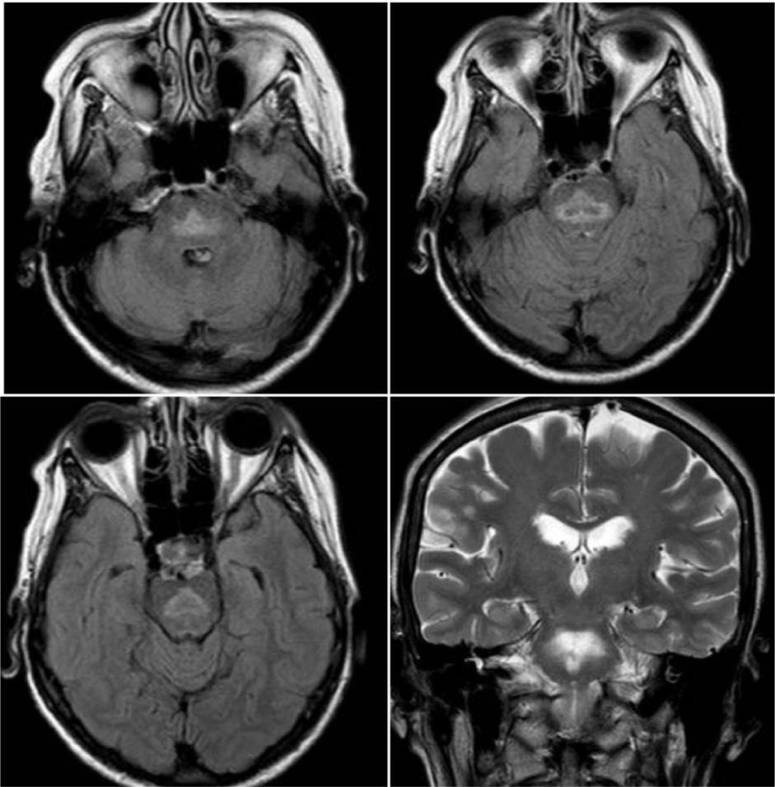
Axial FLAIR MR (a,b,c) and coronal T2 (d) weighted MR showing hyperintensity in the pons.

After hospital discharge, she remained with delusional speech, psychomotor agitation, and loss of functionality. Brain MRI was performed and showed a trident-shaped hyperintensity in the T2- and Flair-weighted sequences in the central portion of the pons (omega sign). These signs can be suggestive of Pontine Myelinolysis (Figure 1).^3^

Currently, the patient presented an improvement in symptoms. She continues to use olanzapine and zolpidem, and is monitored by a neurologist, psychologist, and psychiatrist.

## DISCUSSION

The most interesting aspect in this case was the absence of any characteristic motor manifestations of CPM. Thus, this case illustrates an acute psychiatric disorder in the scenario of correction of hyponatremia being the main symptom of the presentation of CPM, which is a condition rarely described in the literature.

Despite the sodium daily correction level having been performed without exceeding the maximum limit of 12 mMol/L per day, the patient developed acute psychotic symptoms, including confusion, personality change, paranoid hallucinations, tangential speech, echolalia, and reference ideas, which cannot be justified as an infectious manifestation as the screening showed no signs of increase in inflammatory tests or positive cultures. Proper serum sodium correction evolving to CPM is a rare condition but is described in the literature^6^.

The initial brain magnetic resonance imaging performed two days after the onset of psychiatric symptoms (five days after sodium correction) was diagnostic, and showed alterations in the T1, T2, and diffusion on FLAIR-weighted image sequences typically found in CPM^3^.

Psychiatric and behavioral manifestations are unusual clinical presentations in patients with ODS. Psychiatric symptoms usually occur together with the common motor manifestations of CPM. The primary etiology is the demyelination of the ascending fibers of the reticular activator system (SARS) in the pons the primary etiology^1,7^.

The hyperintense signals in the T2-weighted magnetic resonance and the hypointense signals in the T1-weighted magnetic resonance images in areas of pons demyelination are highly sensitive for the diagnosis of CPM. Although MRI is useful in diagnosing ODS, the volume of signal abnormality in T2-weighted MRI has no direct relationship with clinical manifestation^3,6^.

Neurological and neuropsychiatric conditions in CPM have a poor prognostic, and the course of the deficit is commonly considered irreversible. However, some patients may experience partial symptom recovery, while in fewer cases complete recovery can occur. Previous case reports have shown the reversibility of psychiatric symptoms with atypical antipsychotic drugs^8,9^. Therefore, it is recommended that psychiatric manifestations in CPM be managed with atypical antipsychotics and mood stabilizers since the patient is neurologically stable^9–11^.

In conclusion, acute psychosis can appear as the main symptom of CPM, despite being a rare manifestation. Prevention is the key to proper management as there is no adequate treatment, and symptoms can be irreversible in most cases^12^.

## References

[1] Alsaid HM, Naser AM (2019). Central pontine myelinolysis, osmotic demyelination syndrome due to rapidly decreased fluid intake in a schizophrenic patient with psychogenic polydipsia: a case report and review of the literature. J Med Case Rep.

[2] Adams RD, Victor M, Mancall EL (159). Central pontine myelinolysis: a hitherto undescribed disease occurring in alcoholic and malnourished patients. AMA Arch Neurol Psychiatry.

[3] Graff-Radford J, Fugate JE, Kaufmann TJ, Mandrekar JN, Rabinstein AA (2011). Clinical and radiologic correlations of central pontine myelinolysis syndrome. Mayo Clin Proc.

[4] Gupta R, Balhara YPS, Sagar R (2012). Acute psychosis with a favorable outcome as a complication of central pontine/extrapontine myelinolysis in a middle-aged man. J Midlife Health.

[5] Gopal M, Parasram M, Patel H, Ilorah C, Nersesyan H (2017). Acute psychosis as main manifestation of central pontine myelinolysis. Case Rep Neurol Med.

[6] Micieli A, Najeeb U, Kingston W (2020). Central pontine (and extrapontine) myelinolysis despite appropriate sodium correction. Pract Neurol.

[7] Martin RJ (2004). Central pontine and extrapontine myelinolysis: the osmotic demyelination syndromes. J Neurol Neurosurg Psychiatry.

[8] Price BH, Mesulam MM (1987). Behavioral manifestations of central pontine myelinolysis. Arch Neurol.

[9] Lim L, Krystal A (2007). Psychotic disorder in a patient with central and extrapontine myelinolysis. Psychiatry Clin Neurosci.

[10] Mattoo SK, Biswas P, Sahoo M, Grover S (2008). Catatonic syndrome in central pontine/extrapontine myelinolysis: a case report. Prog Neuropsychopharmacol Biol Psychiatry.

[11] Walterfang M, Goh A, Mocellin R, Evans A, Velakoulis D (2012). Peduncular hallucinosis secondary to central pontine myelinolysis. Psychiatry Clin Neurosci.

[12] Biotti D, Durupt D (2009). A trident in the brain, central pontine myelinolysis. Pract Neurol.

